# High-throughput bioinformatics with the Cyrille2 pipeline system

**DOI:** 10.1186/1471-2105-9-96

**Published:** 2008-02-12

**Authors:** Mark WEJ Fiers, Ate van der Burgt, Erwin Datema, Joost CW de Groot, Roeland CHJ van Ham

**Affiliations:** 1 Applied Bioinformatics, Plant Research International, PO Box 16, 6700AA Wageningen, The Netherlands

## Abstract

**Background:**

Modern omics research involves the application of high-throughput technologies that generate vast volumes of data. These data need to be pre-processed, analyzed and integrated with existing knowledge through the use of diverse sets of software tools, models and databases. The analyses are often interdependent and chained together to form complex workflows or *pipelines*. Given the volume of the data used and the multitude of computational resources available, specialized pipeline software is required to make high-throughput analysis of large-scale omics datasets feasible.

**Results:**

We have developed a generic pipeline system called Cyrille2. The system is modular in design and consists of three functionally distinct parts: 1) a web based, graphical user interface (*GUI*) that enables a pipeline operator to manage the system; 2) the *Scheduler*, which forms the functional core of the system and which tracks what data enters the system and determines what jobs must be scheduled for execution, and; 3) the *Executor*, which searches for scheduled jobs and executes these on a compute cluster.

**Conclusion:**

The Cyrille2 system is an extensible, modular system, implementing the stated requirements. Cyrille2 enables easy creation and execution of high throughput, flexible bioinformatics pipelines.

## Background

**Figure 1:**
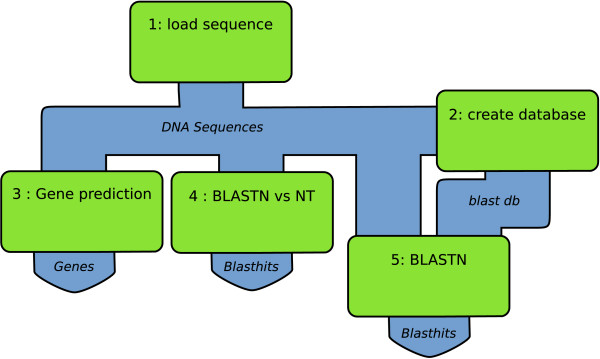
**Simple genome annotation pipeline**. Example of a simple computational pipeline for genome annotation. Green boxes represent nodes. The pipeline describes the execution of a gene predictor (node 3) and two BLAST analyses (nodes 4 and 5) [19] on a set of input DNA sequences (node 1). The BLAST analysis in node 4 compares the incoming sequences against the NCBI NT database. Node 5 uses a BLAST database created by node 2 from the same set of sequences (self-BLAST).

Large-scale computational analysis of biomolecular data often involves the execution of multiple, interdependent operations on an input dataset. The software tools, models and databases that are used in this process need to be arranged in precise computational chains, where output of one analysis serves as the input of a subsequent analysis. Such chains are often referred to as pipelines or workflows. In formal terms, a pipeline can be defined as a graph that describes the order of, and mutual relationships between, the analyses to be performed on an input dataset. In a pipeline representation, an operation performed by a computational tool on input data is represented by a node. The connection between two nodes is represented by an edge and defines a stream of data in-between two analyses. An example of a simple computational pipeline representing part of a genome annotation process is depicted in Figure 1.

Even for a small bioinformatics project with a few interdependent analyses, it is cumbersome to perform all operations manually. For larger projects, e.g. the annotation of a complete eukaryotic genome, which may require the use of dozens of interdependent tools, including gene prediction tools, homology searches against different databases, protein domain analyses and repeat discovery, this quickly becomes excessively complex. The annotation of a genome may require the use of dozens of interdependent tools, including gene prediction tools, homology searches against different databases, protein domain analyses and repeat discovery.

Some of these tools may need to be executed up to tens of thousands of times. The scale and complexity of such computational analyses call for the use of dedicated pipeline software that enables the programming, execution and data-flow management of all analyses required.

With primarily the development of a system for large-scale genome annotation in mind, we have defined the following operational requirements for the design of pipeline management software:

### High-throughput

The system should be capable of handling large datasets, complex data analysis workflows and large numbers of jobs requiring long periods of processing time. To this end, the system must be able to employ a compute cluster.

### Ease-of-use

In a high-throughput data production environment, it is important to have a pipeline system in place that is easy to use by non-expert end-users. This can be achieved by a well-designed graphical user interface (GUI) that allows easy and intuitive creation, adaptation, monitoring and administration of a pipeline.

### Flexible

New or upgraded bioinformatics tools, models and databases appear frequently. To remain up-to-date, it is essential that employment of new or upgraded resources within the pipeline is straightforward. The system should therefore be modular and flexible, and able to accommodate complex data relationships required by some tools. Use of an open communication standard can help to achieve this and ensures the system is compatible with remote resources through the use of web services [1].

### Updates

In ongoing projects it is often undesirable to postpone analysis until all data has been generated. Initial analysis must therefore be repeated on a regular basis, for example, when genome assemblies are updated or new reference data (i.e. BLAST databases) become available. Again, adequate data storage and tracking is important, allowing the pipeline operator to identify the affected parts of the pipeline to reschedule and re-execute only the affected parts of a pipeline with minimal redundancy.

There are a number of pipeline systems publicly available, including Ensembl [2], Pegasys [3], GPIPE [4], Taverna [5], Wildfire [6], MOWserv [7], Triana [8] and Kepler [9]. We will not consider systems that are not publicly available (e.g. the NCBI pipeline). This article describes the development of a new pipeline system, Cyrille2. An obvious question is why we would want to develop yet another system? The answer is, in short, that the available systems do not sufficiently comply with the requirements outlined above. We have built the Cyrille2 system to provide this distinct set of features. In the discussion we will extend the comparison with other systems in more detail.

## Implementation

For a detailed description of the structural design and operation of the Cyrille2 system several key terms must be defined. Table 1 provides the definitions of the most important terms used. Hereafter, we will start with a general overview of the Cyrille2 design, followed by an explanation of how the data-flow is organized within the system. With this knowledge we will continue to describe the core software parts (user interface, Scheduler and Executor) and end with a description of pipeline operation.

**Table 1:** Glossary of most important terms used

**Pipeline**	A pipeline is the definition of a series of computational analyses that are to be performed on a set of data. A pipeline can be described as a graph that is composed of *nodes *which are connected by *edges*.
**Node**	A *node *represents a single analysis in the context of a pipeline. A *node *is associated with a *tool *and is responsible for the execution of one to many jobs. A *node *specifies how the data from a preceding *node *is organized for execution.
**Tool**	A single application embedded in the *pipeline*, for example BLAST.
**Tool-wrapper**	A *tool wrapper *is a script that frames and embeds a *tool *within the *pipeline*. It enables execution of the *tool *through communication with the pipeline software, from which it receives the tools' parameter settings (for example, which BLAST database to use). It translates in- and outgoing data from the pipeline in the format required by the tool.
**Job**	A *job *is a single execution of a *node*. For example, a single gene prediction performed on a DNA sequence loaded into the *pipeline*.
**Edge**	An *edge *connects two *nodes *and describes the *stream *of *objects *that flow between these *nodes*. To allow complex *pipeline *structures, a *node *can define multiple in- and output edges.
**Object**	An object is the most granular element of data traversing the pipeline. Each object is tracked by the Cyrille2 system.
**Stream**	A *stream *is a serie of objects traversing an *edge *between two *nodes*.

### System overview

**Figure 2:**
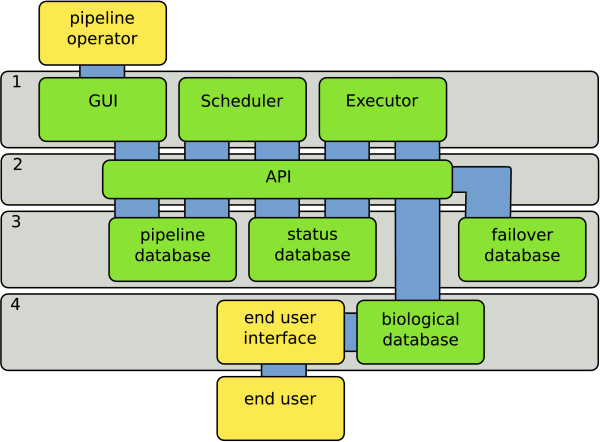
**The Cyrille2 system architecture**. Main architectural layers are numbered (1–4). See the text for further description.

The Cyrille2 system architecture is composed of four distinct layers (Figure 2). Layer 1 comprises the main functional and core software components. These core components make extensive use of a modular application programming interface (API) (layer 2). The API allows unified access to three system databases (layer 3). The biological database and the end-user interface that connect to it are third-party systems that can be integrated with the Cyrille2 system (layer 4). To allow tracking and debugging of a pipeline in operation, a centralized status and logging system is implemented. This provides a pipeline operator access to detailed information on the status of a pipeline run and errors that might have occurred.

A pipeline system needs to manage and store large amounts of diverse information. To keep different types of data separated, the system employs four databases (Figure 2, layers 3 and 4): (1) the pipeline database which stores pipeline definitions, node settings and associated parameters; (2) the status database which stores the execution state of a pipeline at any given time, tracking all jobs and their respective in- and output.; (3) the biological database which stores and provides access to the results of all analyses and; (4) a failover database which employs a generic method to store all data that does not need to be stored in the biological database.

Consider, as an illustration, the gene-prediction node (node 3) from the example pipeline given in Figure 1. The pipeline database identifies this node as a gene prediction node and stores all instructions (i.e. tool name and parameters settings) on how the gene prediction tool is to be executed on each input DNA sequence from the preceding Load sequence node (node 1). The status database stores information on which of the input sequences the gene prediction has been performed, which genes have been predicted and tracks all objects associated to this analysis in the biological database using unique object identifiers.

**Figure 3:**
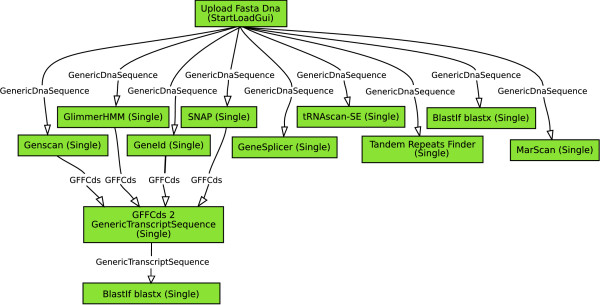
**Screenshot of a pipeline created with the Graphical User Interface**. A simple genome annotation pipeline is shown, consisting of four gene prediction analyses (GlimmerHMM, Genscan, GeneID and SNAP), an intron-exon splice-site prediction (GeneSplicer), a tRNA gene predictor (tRNAscan-SE), a MAR element scan (Marscan), a repeat analysis (Tandem Repeat Finder) and a BLASTX analysis. The predicted genes (stored as coordinates on the original sequence; GFFCds) are converted into a sequence object (GenericTranscriptSequence) and subsequently subjected to a BLASTX analysis against the NCBI NT database.

Similar to the functional division of the databases, the core software is divided into three distinct functional parts: the Graphical User Interface (GUI), the Scheduler and the Executor (Figure 2, layer 1). The GUI allows a pipeline operator to create, adapt, start and stop pipeline runs and fine-tune pipeline and tool settings. A screenshot of a genome annotation pipeline created with the GUI is given in Figure 3. Additional series of screenshots showing the operation of a small pipeline using the GUI are given in Additional file 1.

The Scheduler is the core of the Cyrille2 system. It retrieves pipeline definitions from the pipeline database and schedules all jobs for execution, accounting for dependencies between nodes. A scheduled job is stored in the status database. Further details on Scheduler operation are given below. The Executor loops through all scheduled jobs and executes each of these. The results of each job are stored in the biological database and are tracked with unique object identifiers in the status database. If the number of jobs to be executed is large, a compute cluster is required to keep the total execution time within bounds. To this end the Executor acts as a broker between the Cyrille2 system and third-party compute cluster software such as Sun Grid Engine (SGE). It is possible to employ multiple and different types of clusters by running multiple instances of the Executor.

**Figure 4:**
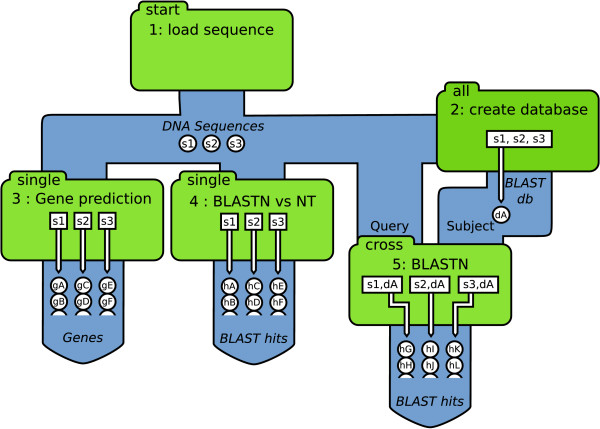
**Data flow within Cyrille2**. Detailed illustration of the data flow and scheduling strategies of the node types from the pipeline shown in Figure 1. Green boxes represent nodes. The different node types start, single, all, and cross are indicated in the top left corner of each box. In this example, three DNA sequences are uploaded into the system (s1, s2 and s3) which are subsequently processed by the different nodes. Open circles in-between the nodes indicate objects traversing the pipeline. Open boxes inside the nodes represent the jobs that are scheduled. For example; sequences uploaded (s1, s2 and s3) are scheduled by node 4 for a BLASTN analysis against the NT database. This BLAST analysis results in the BLAST-hits indicated by objects hA-hF. See text for more details.

Whereas the Cyrille2 pipeline software functionally consists of three separate parts, from a software implementation point of view, the essential application logic of the system is implemented using object oriented programming. The system implements different scheduling strategies in different node types (Figure 4). A new node type (i.e. a node class) can be created from scratch or it can inherit from an existing node class making the implementation of new functionality as easy as possible.

### Data flow and storage

A major challenge for any pipeline system is to devise a fast and robust way to conduct data through a pipeline. This is not trivial, given that even a relatively simple pipeline (such as the one given in Figure 1) may imply that many thousands of separate jobs need to be scheduled and executed, which in turn may result in millions of objects.

Automated execution of a pipeline implies that each node needs to hold information on the nature and format of the objects that enter and leave it and that it has to process such streams in a manner unique for each type of data. For example, a stream of DNA sequences is different from a stream containing BLAST reports. The issue is best approached using a uniform syntax and identification of the data transported in a pipeline. This does not only allow for a standardized implementation of the scheduling strategies of different node types, but also for a generic node interface description and for data tracking.

Several data exchange formats, with varying scope, have been devised and proposed for the handling and communication of biomolecular data, including XML-based formats such as GAME (used by the Apollo genome annotation curation tool [10]) and BioMOBY [11], and flat-file formats such as GFF. An appropriate data exchange format identifies and communicates data in a uniform and unambiguous manner. Such a format must permit unique identification and classification, and it must be extensible to accommodate future incorporation of novel data types. With this in mind, we have chosen to implement BioMOBY [11] as data exchange format for the Cyrille2 system. BioMOBY is emerging as an important data standard in bioinformatics and is already used by MOWserv [7] and Taverna [5] when this system is dealing with BioMOBY operations.

The BioMOBY standard contains a specification on how to describe data types, formats, and analysis types. It is a meta-data format, meaning that it does not describe data but defines how to describe data. BioMOBY employs a system of object identification and classification, in which each BioMOBY object is identified with (1) an identification string (id), (2) an object type (articlename) and (3) a namespace. BioMOBY encompasses the description of web services and facilitates interoperability with third-party servers. In the current era of distributed computing, this ability to communicate with systems worldwide is becoming ever more important.

**Figure 5:**
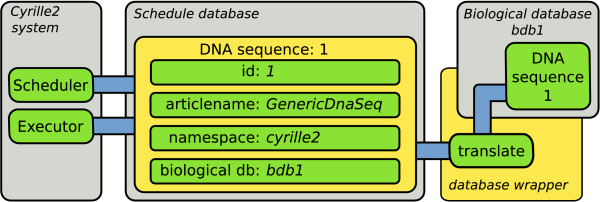
**Relationship between the status and the biological database**. The status database employs BioMOBY identification (id, articlename and namespace) and holds information on the biological database in which the object is stored. The biological database is accessed through a database specific wrapper that provides a generic interface to retrieve an object based on the information in the schedule database. The database wrapper is accessed through a wrapper script (number 1 and 3 in Figure 6).

Standardized object identification is also applicable in standardized data storage. With a BioMOBY object stored with a unique id (as provided by the Cyrille2 system), articlename and namespace, these three values are sufficient to uniquely retrieve the object from a database. The Cyrille2 system is designed to allow the use of different databases schemas and/or engines to ensure flexibility. A database wrapper functions as an intermediate between the object identity on the one hand and database-specific storage and retrieval of these objects on the other hand (Figure 5). The database wrapper contains specific instructions to store, retrieve and delete each different object type in the biological database. This solution combines the unique identification of any object with the freedom to use any database. The database wrapper currently implemented in our system is written for the Generic Genome Browser database schema using MySQL. Two other database schemes have been implemented for use in projects on miRNA discovery and comparative genomics.

Storage of all intermediate data generated during pipeline operation is guaranteed by the failover database that automatically stores any object not stored in the biological database. This may happen for two reasons: firstly, an intermediate object might be of no importance to the end-user querying the biological database, or, secondly, the database wrapper fails to store the object to the biological database for an unexpected reason; in either case, no data is lost.

Apart from transporting data in between separate nodes of the system, a pipeline system needs methods to upload new data into the system and retrieve the results afterwards. To upload data into the Cyrille2 system, specific start nodes are provided allowing the upload of data through the user interface or automatically harvesting data from a file system. The resulting data of the pipeline is stored in a domain specific database, for example the Generic Genome Browser database [12], which is commonly used in genome annotation. The web interface is, in this case, a bonus that helps end-users to access the data.

### Scheduler

The Scheduler is the core of the Cyrille2 system. Based on a pipeline definition (from the pipeline database) it schedules all jobs for execution, taking mutual dependencies between nodes into account. Various tools used in an analysis pipeline require different arrangements of incoming data. For example, node 2 in Figure 1 uses all DNA sequences from node 1 to create a BLAST database. The Scheduler thus arranges all sequences to be processed by a single job. In contrast, node 4 processes each sequence separately in a BLAST analysis. In this case the Scheduler creates as many jobs as there are input sequences. This is illustrated in Figure 4, which shows the same pipeline as given in Figure 1, but now expanded with detailed information on the objects that are created and the jobs that are scheduled.

Scheduler functionality is embedded in the node classes. This modular, object-oriented implementation of a node allows for complex scheduling strategies. A more complex node implemented in the Cyrille2 system schedules groups of objects which share a common grandparent, for example, all repeats that are predicted by several different repeat detection tools, grouped per BAC sequence (the grandparent).

### Pipeline execution

Execution of a pipeline can be considered at two levels: execution of a separate node, and execution of an entire pipeline. A single node in the Cyrille2 system executes a variable number of distinct jobs. For example, a BLAST analysis of 10 input sequences requires a BLAST node to execute 10 BLAST jobs. A single BLAST job consists of several processing steps: retrieve the input sequence (in BioMoby format) from the biological database; export the sequence as a FASTA file; execute BLAST with the correct parameters; read and parse the resulting BLAST report to a BioMOBY representation; write the BioMOBY formatted BLAST report to the biological database, and; register the results in the status database.

**Figure 6:**
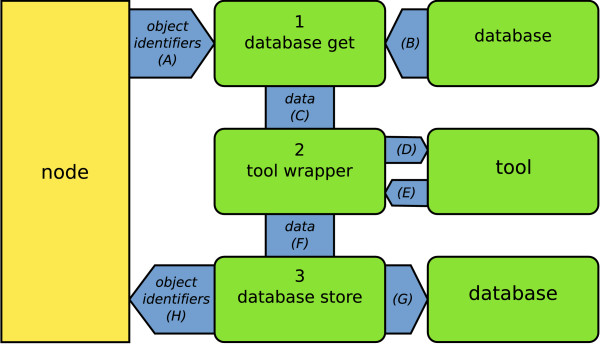
**Execution and data flow in a node**. The standardized execution and data flow within a separate node in the Cyrille2 system. Execution starts with sending the object identifiers describing which objects serve as input for this specific node (A). The database-get script retrieves this data from the biological database (B), converts it to BioMOBY format (C) and sends it to the tool wrapper. The tool wrapper prepares the data for the tool (D), executes the tool, interprets its output (E) and converts it to BioMOBY (F). The database-store script stores the data in the biological database (G) and returns the newly created object identifiers (H) back to the system.

Node operation in Cyrille2 is performed by executing three different scripts (Figure 6): (1) data is retrieved from the database; (2) the tool is executed, and; (3) the results are stored back in the database. Communication with the database is handled by two database connection scripts (steps 1 and 3, see also Figure 6: database-get and database-store), which is equivalent to the Ensembl RunnableDB [13]. These two scripts access the database wrapper (Figure 5) and provide generic communication with any database of choice.

A tool wrapper is responsible for the execution of the tool and provides generic interaction with the Cyrille2 system. Tool wrappers are implemented in such a way that they can run standalone, be part of a BioMOBY web service, or function as a component of the Cyrille2 system. A tool wrapper is equivalent to a Runnable in the Ensembl system [13]. During execution, the tool wrapper is responsible for steps D, E and F from Figure 6.

A further task of the tool wrapper is to register itself in the Cyrille2 system. Registration implies that the tool becomes available through the GUI, allowing a pipeline operator to integrate it into a pipeline and allowing the Scheduler to correctly schedule jobs for that tool. The process communicates what type of objects are required as input (e.g. protein sequences for BLASTP), what parameters are accepted (e.g. specification of a protein database) and with what node type it must be associated. This is implemented in a generic registration method where the wrapper registers all required information into the pipeline database.

In a rapidly evolving field like bioinformatics, it is of great importance that new tools can be implemented quickly. In the Cyrille2 system this requirement is implemented through modular, object oriented, design of the tool wrapper code. In brief, implementation of a novel tool in the Cyrille2 system involves the following procedure: (1) installation and configuration of the new tool on the execution server or cluster; (2) writing of the BioMOBY-compatible tool wrapper; (3) definition of new BioMOBY objects (if required); (4) confirmation of compatibility between object types and the biological database in use, and; (5) registration of the tool in the pipeline database.

A complete pipeline operates by iteratively running the Scheduler and Executor. Results produced by a tool under control of the Executor can result in more jobs to be scheduled by the next Scheduler run. If there are no more jobs to be executed for a node and all its parents, it is flagged as finished in the status database. A complete pipeline is finished if all nodes are in the finished state. Pipeline iteration can be resumed after new data is uploaded into the pipeline, when a database has been updated (e.g. BLAST databases) or when the pipeline definition has changed. Resumption is accomplished by unflagging the finished state of one or more nodes in a pipeline. This is either done manually (through the GUI) or automatically, for example after a BLAST database update.

### Results

Our local implementation of the Cyrille2 system runs on a dedicated server (dual AMD Opteron 850, 4 Gb memory, 300 Gb disk) and has a 50 CPU, SGE based Linux compute cluster at its disposal. A list of third party tools currently wrapped in the Cyrille2 system is provided [see Additional file 2].

In a test run, the Cyrille2 system analyzed 50 Arabidopsis BAC sequences (4.8 Mb) randomly downloaded from NCBI using the pipeline shown in Figure 3. The analyses resulted in over 735.000 objects created in over 10.000 different analyses executed. The results are summarized Additional file 3. Measurement of the pipeline execution time is not relevant as the bulk of the execution time results from executing the actual tools. As an illustration, however, the analysis of a single BAC with the pipeline from Figure 3 takes approximately an hour to complete on a Linux compute cluster with 50 CPUs (hardware specifications as above).

The Cyrille2 system is now used routinely for BAC annotation in two solanaceous genome sequencing projects in which our group is involved [14,15]. In addition, we run the system in a comparative genomics project of fungal genomes and a second project on the large-scale prediction of microRNAs in plant and animal genomes. These last two projects require very high data throughput and employ databases different from the Generic Genome Browser database and thus demonstrate the flexibility of the Cyrille2 system and its ability to execute complex and computationally demanding pipelines.

## Discussion

The Cyrille2 system was developed with the aim of providing an automated, high-throughput, easy-to-use, flexible and extensible bioinformatics workflow management system. Among its most notable features are the implementation of a powerful job scheduler module, storage of intermediate data, compatibility with different database types for storage of biological data, a generic tool wrapper module, and uniform data transport and data tracking.

Ease-of-use is achieved through implementation of an intuitive user interface with several layers of complexity. A pipeline operator can select from a predefined set of pipelines and nodes to perform complex data analysis tasks while an administrator is able to construct novel, and fine-tune existing, pipelines.

### High-throughput operation

The major part of the development effort has been directed towards achieving flexibility and extensibility in architecture and high-throughput operation. In a high-throughput data analysis environment, parallel execution of jobs is important to optimally use the available computational facilities and hence, make pipeline calculation time as short as possible. This requires specific scheduling logic for different node types. The Cyrille2 Scheduler prepares jobs for parallel execution as soon as results from preceding analyses become available. The single node type schedules a job immediately after an input object becomes available. This means that subsequent analyses can already start before the parent node is finished. Most pipeline systems implement a scheduling engine able to schedule jobs in parallel [3-9,12]. An important feature of Cyrille2 is the modular implementation of the node class allowing a greater variety in scheduling strategies.

Parallel scheduling requires parallel execution, which is controlled by the Executor. There are many solutions available for the distribution of jobs over a compute cluster, including Sun Grid Engine (SGE), Condor, OpenPBS and LSF. For the Cyrille2 Executor, we have chosen to employ SGE, which is both stable and able to handle high loads. A port for Grid technologies such as Condor is under development and will allow the Cyrille2 system to employ idle Windows desktops.

Another important aspect in high-throughput pipeline analysis is the storage of intermediate results. If this is implemented, the pipeline system will be able to resume calculations close to the point where it may have stopped after a system failure. This feature becomes important when a pipeline requires a long execution time and hence, the chance of a failure, somewhere in the system, increases. If storage of intermediate data is undesirable, for example because of disproportional usage of storage capacity, it is straightforward to either develop a node type which embeds two or more other nodes and directly transfers the data between the nodes in a single Executor run, or to develop a single tool wrapper which executes both steps and behaves as a single tool in the system. In both cases, intermediate data storage is by-passed.

A further advantage of intermediate data storage is that each part of a pipeline can be re-executed when necessary. This is essential when only part of a pipeline needs to be repeated with either different parameter settings, after a database update, or upon the addition of extra nodes to the pipeline. In the current implementation of Cyrille2, the system will remove, prior to a rerun, all data that is affected by the update from both the status and biological databases and rerun the necessary analyses. For example, consider the genome annotation pipeline of Figure 3. Prior to uploading a new version of a sequence, the pipeline operator will flag this sequence, instructing the system to delete all gene predictions and BLAST hits associated with that sequence. The system will subsequently perform only those analyses on which the changed sequence had an impact.

We have encountered two severe problems during Cyrille2 development, both related to system overhead: 1) BioMOBY XML parsing is very time-consuming for large data-sets, and; 2) SGE overhead becomes very large for nodes which execute a large amount of small jobs. The import and export of large XML files in general is notoriously slow. In Cyrille2, we have solved this problem by circumventing raw XML transport as much as possible. For example, conversion of the data between steps 1, 2 and 3 in the node depicted in Figure 6, from raw XML to an internal python object representation of the BioMOBY XML boosted the performance of this node significantly. Using serialized objects might, as a drawback, have an impact on backwards compatibility, specifically if objects are stored in the Failover database.

The second major obstacle concerned the overhead involved in executing large numbers of jobs with very short computation time. If each of these jobs is scheduled separately on an SGE cluster, the overhead used by SGE considerably exceeds the time required for the execution itself. This overhead was significantly reduced by implementing a generic batch mechanism which is able to execute an arbitrary amount of pipeline jobs as a single SGE job.

### Flexibility

In the rapidly evolving field of genome annotation, it is critical that a pipeline management system is flexible and easily extensible. The Cyrille2 system was designed to allow future incorporation of novel tools, data types and databases in a generic fashion. For example, for a present-day genome annotation project, it is generally sufficient to store all relevant data in a biological database such as the Generic Genome Browser database [12]. However, if one would require the inclusion of data such as multiple alignments or 3D protein structures, a different database is required.

The Cyrille2 system is designed to make the addition of a novel object type or the complete change of the biological database as easy as possible. This is achieved by implementation of the database wrapper as a separate module. Addition of a novel data type can be done by adding a 'get', 'store' and 'delete' function for this type of data type to the database wrapper. To create a wrapper for another database schema, a new module must be written with a storage, retrieval and delete function for each object type. This mode of integration of a third-party database with a pipeline system is unique for the Cyrille2 system. Many alternative systems do not use a database for storage of intermediate results, (Taverna [5]; GPIPE [4]; Wildfire [6]). Instead, these systems transport the output of one program directly to the next program and/or store intermediate results as flat files. Such an approach is unsuitable for large-scale data analysis. A database system is better suited to keep track of many, possibly millions, of intermediate objects and their mutual relationships. Moreover, a database is better adapted to distribute data in a heterogeneous environment. Other systems do employ a database for data storage (Ensembl [13]; Pegasys [3]; MOWserv [7]) but each of these is strongly linked to a specific database schema, thus limiting their flexibility.

In the current implementation of Cyrille2, the Generic Genome Browser database [12] is used for storage of the genome annotation data with, as an obvious extra, the Generic Genome Browser allowing an end-user easy access to the annotations. Implementing Cyrille2 in different projects, such as comparative genomics and miRNA prediction has proven the capability of Cyrille2 to operate using different databases schemes.

The use of BioMOBY as a communication standard combined with the storage of standardized object identifiers by the Cyrille2 system ensures that any object can be handled and tracked by the system, including binary objects such as images [11]. Other advantages of using BioMOBY are that it ensures easy integration with the growing body of remote BioMOBY web services and optimal interconnectivity between nodes. At the same time, it is possible for external users to access the Cyrille2 tools using any BioMOBY client if the tools and toolwrappers are placed, with some minor modifications, on a web server.

Several systems employ specific embedded scripts to translate the output of one tool to the input of the next (Wildfire, [6]; FIGENIX [16]; GPIPE [4]) as opposed to using a standardized data format. Most analysis tools have a unique in- and output format and thus the number of unique translation steps grows quickly with the number of tools wrapped. This can be mitigated by using uniform data transport such as BioMoby, which is used in Cyrille2, MOWserv [7] and Taverna [5] when this system is dealing with BioMOBY operations. The Ensembl system employs a uniform Perl data structure to the same end [17].

On a higher level, import and export of the description of a complete pipeline would improve flexibility by allowing the exchange of workflows with other systems. For example, a pipeline developed in Taverna could then be executed in Cyrille2, or vice versa. There are several candidate languages available such as Scufl, used by Taverna, or the more widely accepted BPEL [18]. Research is necessary to see if the adaptation of such a standard would be worthwhile for the Cyrille2 system, both to see if any candidate provides sufficient flexibility as to assess the amount of work necessary to implement such a standard.

### Conclusion

The Cyrille2 system has been developed to operate in the environment of a high-throughput sequencing facility with a need for robust, automated and high-throughput genome analysis, and easy creation, adaptation and running of pipelines by non-expert users.

Most of the pipeline systems that have recently been released were developed as a workbench for bioinformaticians. Some systems excel in the way they allow for complex pipelines to be built through a visually appealing but sometimes complex GUI (Taverna, Kepler, Triana). Most systems are not suited for automated, high-throughput operation with as obvious exception Ensembl [13]. Ensembl was, however, not designed to be deployed at other sites or execute *ad hoc *pipelines.

In view of the distinctive functionality and combination of features implemented in the Cyrille2 system we believe that it is a valuable addition to the array of pipeline systems available and particularly useful in environments that require high-throughput data analysis. In the near future we are planning to expand the Cyrille2 system in computational workflows for metabolomics data analysis.

## Availability and requirements

Cyrille2 is written in Python on a Linux platform and requires a MySQL database server and an Apache/mod_python web server. The system expects a Rocks Linux cluster for execution of analysis tools, although other SGE based solutions should work. The source code of the Cyrille2 system is published under the terms and conditions of the GNU Public License and is freely available from .

## Authors' contributions

MF is the lead developer of the Cyrille2 system, has implemented most of the core of the system and has drafted this publication. AvdB and ED have both contributed to the development of tool wrappers, the database layer, limited work on the core and the manuscript. JdG has aided in implementing the GUI. RvH has been the project manager and contributed in discussions, planning and writing of this manuscript. All authors have read and approved the manuscript.

## Supplementary Material

Additional file 1 Fiers.Cyrille2.Suppl1.pdf contains a series of screenshots showing the creation and operation of a small pipeline using the Cyrille2 system. Click here for file

Additional file 2 Fiers.Cyrille2.Suppl2.pdf contains a list of tools currently wrapped for use in the Cyrille2 system. Click here for file

Additional file 3 Fiers.Cyrille2.Suppl3.pdf contains the results of a test run with the pipeline from Figure 3 and 50 randomly downloaded BACs. Click here for file
