# Supplementary material for: High-throughput bioinformatics with the Cyrille2 pipeline system
Source: BMC Bioinformatics. 2008 Feb 12;9:96. doi: 10.1186/1471-2105-9-96 (PMC2268656; doi:10.1186/1471-2105-9-96)
Supplement: Additional file 1 — Fiers.Cyrille2.Suppl1.pdf contains a series of screenshots showing the creation and operation of a small pipeline using the Cyrille2 system. [file 1471-2105-9-96-S1.DOC]

Supplementary Information 1 to:

# High-throughput bioinformatics with the Cyrille2 pipeline system

### Mark WEJ Fiers^1^, Ate van der Burgt^1^, Erwin Datema^1^, Joost CW de Groot^1^, Roeland CHJ van Ham^1§^

^1^Applied Bioinformatics, Plant Research International, PO Box 16, 6700AA Wageningen, The Netherlands

^§^Corresponding author

## Operation of the Cyrille2 system

This document contains a series of screenshots which represent the creation and operation of a small example pipeline in the Cyrille2 pipeline system. The pipeline in this example starts with the upload of a number of BAC sequences into the system. Subsequently three gene predictions are performed on each of the input sequences.

The first section of the interface that is usually visited during the operation of the Cyrille2 system is called the ‘All Pipelines’, and contains an overview of the pipelines present in the system, as shown by the screenshot in Figure 1. A new pipeline can be created through the “pipeline / Add a new pipeline” menu.

#
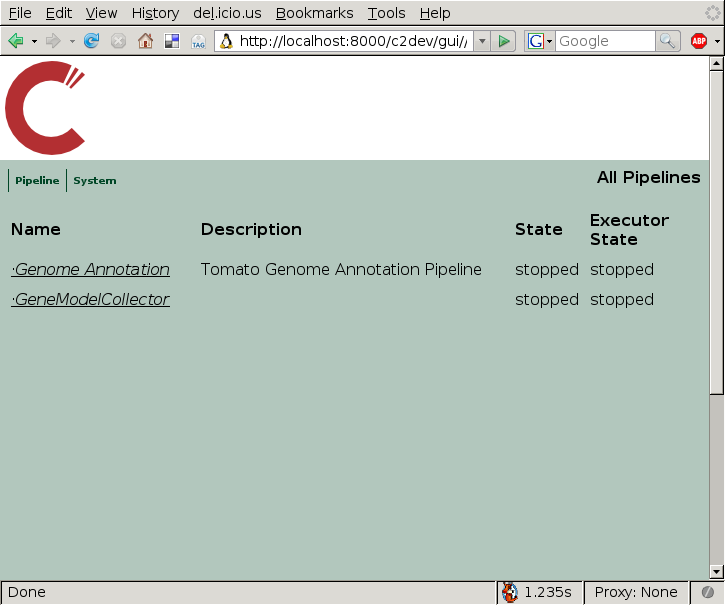


*Figure 1: The ‘Pipeline Overview’ view of the graphical user interface presents a list of all available pipelines in the Cyrille2 system.*

After creating a new pipeline, the pipeline can be built or altered in the ‘Pipeline Edit’ view, accessible by clicking on the name in the ‘All pipelines’ view. A pipeline which has been newly created from the ‘Pipeline’ menu only contains a ‘Start Node’ (see Figure 2). This node does not represent any analysis in the pipeline, but rather serves as the root of the pipeline.


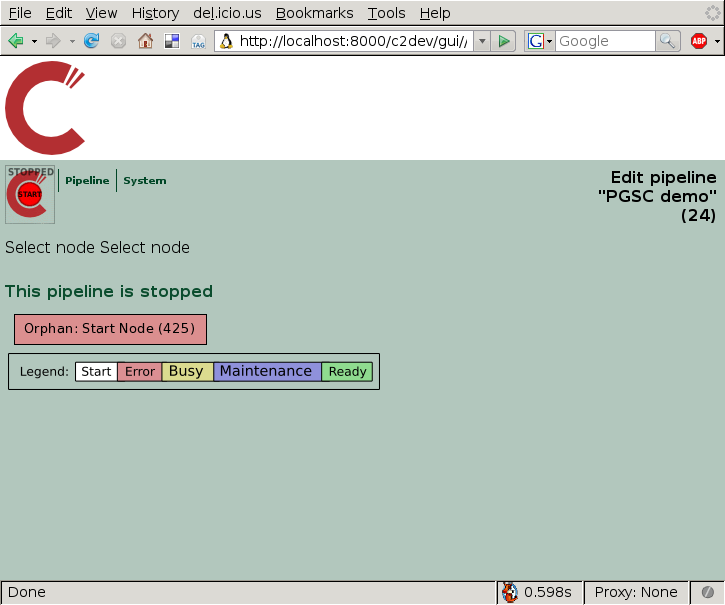
*Figure 2: A newly created pipeline as displayed through the ‘Pipeline Edit’ view. The pipeline contains a single, unconnected (‘Orphan’) Start Node, which functions as the root of the pipeline.*

In order to add nodes to the pipeline, the Start Node must be selected. Nodes can only be added as children of another node, illustrating the necessity of the Start Node. After selecting the Start Node (by clicking on it), the ‘Node edit’ menu appears in the top right corner of the ‘Pipeline Edit’ view (see Figure 3). This menu is used to add new nodes to the pipeline. Selecting the ‘Add a child’ select-box unfolds a list of nodes that can be added as a child of the currently selected node (in this case, the ‘Start Node’). This list contains only the nodes that can serve as valid child nodes to the currently selected node. In the case of the ‘Start Node’, only nodes that can upload data into the system are considered valid. In Figure 3, an ’Upload Fasta DNA’ node is added to the ‘Start Node’ of the pipeline.


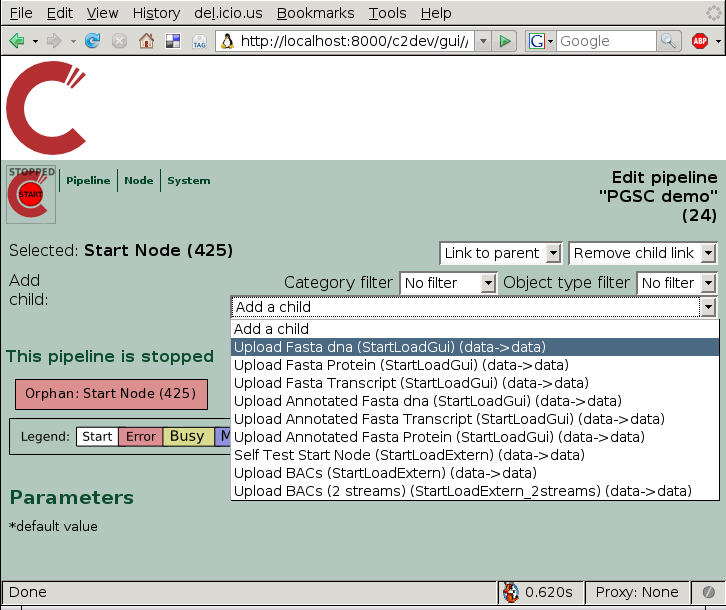


*Figure 3: Adding an ‘Upload Fasta DNA’ node to the ‘Start Node’ of the pipeline.*

After confirming the creation of the selected child nod, it appears in the diagram in the ‘Pipeline Edit’ view (see Figure 4). Since this node requires user input to upload a sequence, it is marked as such.


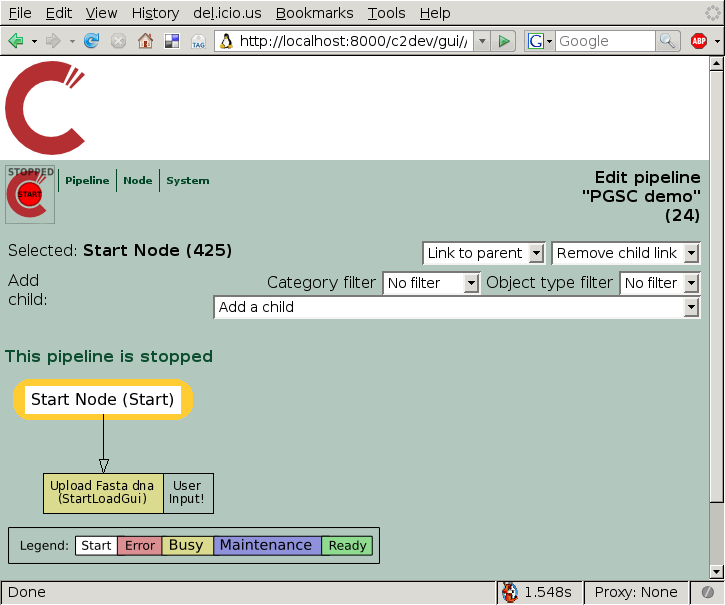
*Figure 4: Pipeline diagram from the ‘Pipeline Edit’ view, showing the addition of the ‘Upload Fasta DNA’ node to the pipeline. The label ‘User input!’ that is attached to the ‘Upload Fasta DNA’ node indicates that this node requires manual intervention from the user (in this case, the upload of one or more Fasta DNA sequence files).*

In order to use the newly added node to upload BAC sequences into the system, the ‘Upload Fasta DNA’ node must be selected by clicking on it. After selecting the node, an “upload file” option has appeared in the menu on the top right of the ‘Pipeline Edit’ view (see Figure 5). To upload one or more sequences into the system, the user can select a Fasta file on disk using the ‘Browse...’ and ’upload’ button.


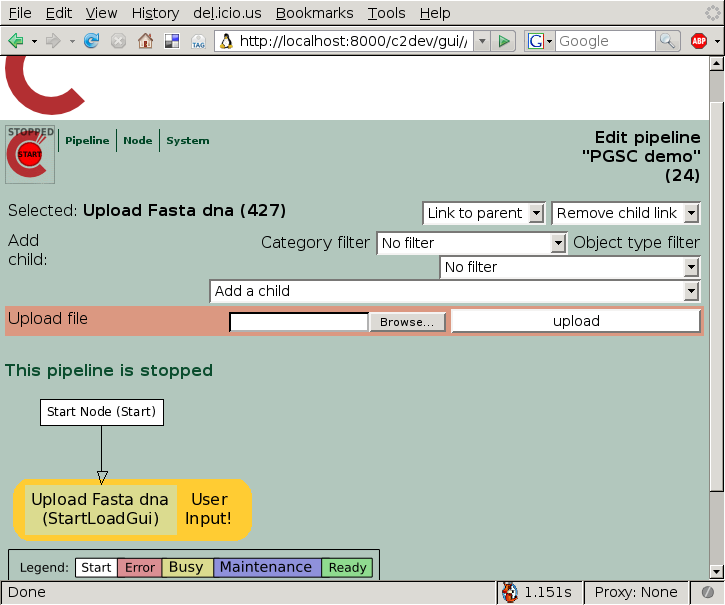
*Figure 5: Uploading a Fasta file into the system using the ‘Upload Fasta DNA’ node*.

Addition of nodes that perform analyses on the uploaded sequences can be performed in a manner similar to the addition of the ‘Upload Fasta DNA’ node. That is, the user selects the ‘Upload Fasta DNA’ node, and selects a node to be added from the ‘Add Child’ menu. Since the ‘Upload Fasta DNA’ node produces ‘GenericDnaSequence’ objects from the Fasta file it receives as input, only the nodes require a ‘GenericDnaSequence’ object as input can be added to this node. In Figure 6, a “Genscan” analysis has been added to the pipeline. When this node is selected, a list of parameters that can change the behavior of this node is presented below the pipeline diagram in the ‘Pipeline Edit’ view.


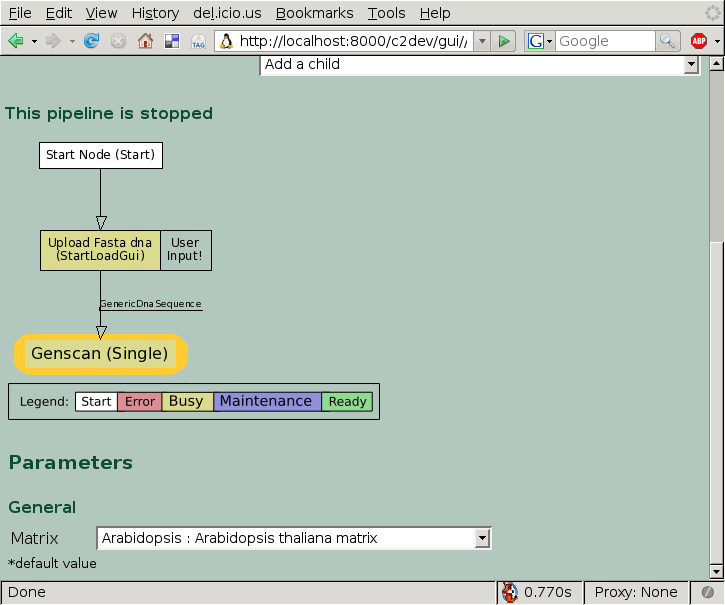
*Figure 6: Addition of a ‘Genscan’ node to the pipeline. The ‘Genscan’ node thakes the ‘GenericDnaSequence’ objects that have been created by the ‘Upload Fasta DNA’ node as input, and performs a genscan analysis (a gene prediction) on each of the input sequence objects. Below the pipeline diagram, the available parameters for the ‘Genscan’ node are presented. In this case, there is only one parameter that can be edited by the user.*

At any time it is possible to check the status of the pipeline by selecting the ‘Status’ option form the ‘Pipeline’ menu. Figure 7 depicts the ‘Status’ view of the previously created pipeline. From the status view, it can be gathered that 16 BAC sequences were uploaded into the system, and that an identical number of jobs have been scheduled for the Genscan node. Figure 7 shows the status of the pipeline, after the ‘Genscan’ node has finished its analysis on each of the 16 sequences. Here, the ‘Genscan’ node has produced 86 output objects, which together represent the genes predicted by Genscan.


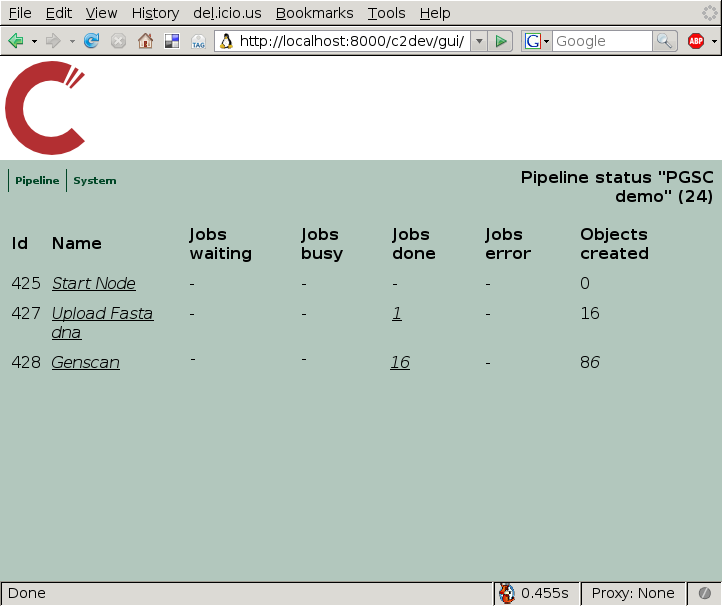
*Figure 7: ‘Status’ view of the pipeline after performing the Genscan analysis on each of the 16 input sequences.*

At any time the pipeline can be expanded to include more nodes. In Figure 10, two additional nodes (namely, a ‘GlimmerHMM’ and a ‘BlastIf blastn’ node) have been added to the pipeline, using the same method as described for adding the ‘Genscan’ node. The system subsequently performs these analyses on the BAC sequences without repeating the previously completed Genscan analyses*.*


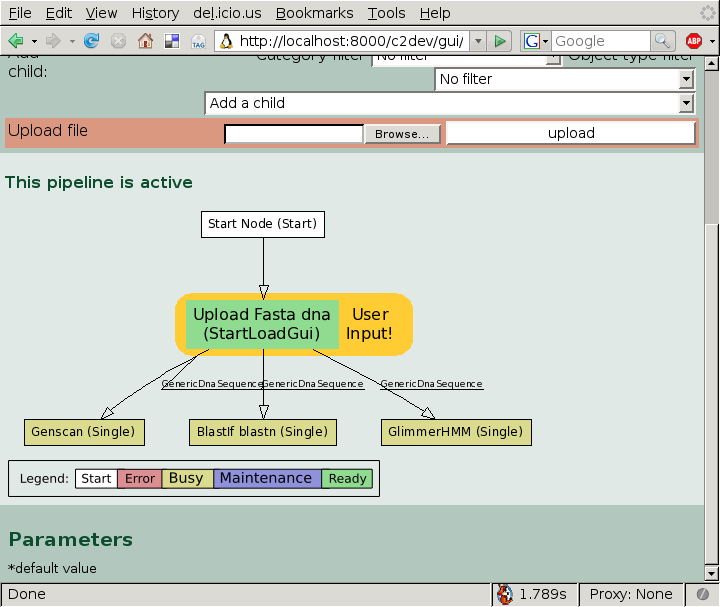
*Figure 8: Addition of a ‘GlimmerHMM’ and a ‘BlastIf blastn’ node to the pipeline.*

Of course, the example shown here presents a simple pipeline on a very small data set. The ‘Status’ view of a more realistic BAC annotation pipeline shows that the Cyrille2 system can indeed handle a large number of objects (see Figure 8).


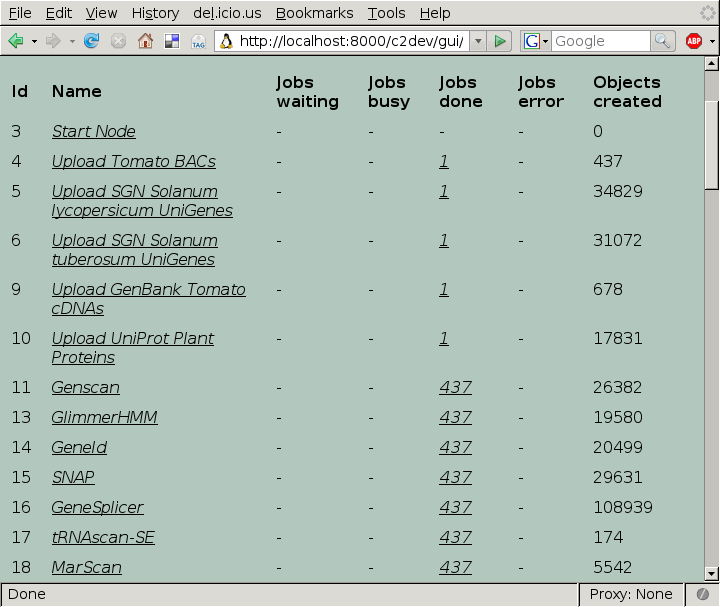


*Figure 8: ‘Status’ view of a BAC annotation pipeline in a production environment. This pipeline was used for the in-depth annotation of 437 tomato BAC sequences. Note that the screenshot only shows the status of 13 nodes of the pipeline, whereas the complete pipeline contains 33 nodes.*
