# Supplementary material for: High-throughput bioinformatics with the Cyrille2 pipeline system
Source: BMC Bioinformatics. 2008 Feb 12;9:96. doi: 10.1186/1471-2105-9-96 (PMC2268656; doi:10.1186/1471-2105-9-96)
Supplement: Additional file 2 — Fiers.Cyrille2.Suppl2.pdf contains a list of tools currently wrapped for use in the Cyrille2 system. [file 1471-2105-9-96-S2.DOC]

Supplementary Information 2 to:

# High-throughput bioinformatics with the Cyrille2 pipeline system

### Mark WEJ Fiers^1^, Ate van der Burgt^1^, Erwin Datema^1^, Joost CW de Groot^1^, Roeland CHJ van Ham^1§^

^1^Applied Bioinformatics, Plant Research International, PO Box 16, 6700AA Wageningen, The Netherlands

^§^Corresponding author

## Tools currently wrapped for use inside Cyrille2

List of tools currently wrapped for use in the Cyrille2 system. Only third party tools are listed; tools for internal use, such as data conversion tools, are omitted.

| **Application** | | **Reference** | **Application** | | **Reference** |
| --- | --- | --- | --- | --- | --- |
| Similarity / homology search tools | | | | | |
|  | BLAST | [1] |  | BLASTIF | in house  developed |
| Gene prediction tools | | | | | |
|  | Genscan | [2] |  | GeneID | [3] |
|  | GlimmerHMM | [4] |  | SNAP | [5] |
|  | Glimmer | [6] |  | Augustus | [7] |
|  | GeneSplicer | [8] |  | GetOrf (EMBOSS) | [9] |
|  | Jigsaw | [10] |  |  |  |
| Alignment | | | | | |
|  | Clustalw | [11] |  | Sim4 | [12] |
|  | GeneWise | [13] |  | Mummer | [14] |
| Other | | | | | |
|  | Tandem Repeat Finder | [15] |  | InterPRO | [16] |
|  | tRNAscan-SE | [17] |  | Marscan (Emboss) | [9] |
|  | RNAfold | [18] |  | TribeMCL | [19] |
|  | Inparanoid | [20] |  | RepeatMasker | [21] |
|  | TGICL | [8] |  |  |  |

References

1. Altschul, S. F., Gish, W., Miller, W., Myers, E. W. & Lipman, D.J.: Basic local alignment search tool.. J Mol Biol 1990, 215:403-10.

2. Burge, C. & Karlin, S.: Prediction of complete gene structures in human genomic DNA.. J Mol Biol 1997, 268:78-94.

3. Guigó, R., Knudsen, S., Drake, N. & Smith, T.: Prediction of gene structure.. J Mol Biol 1992, 226:141-57.

4. Majoros, W. H., Pertea, M. & Salzberg, S.L.: TigrScan and GlimmerHMM: two open source ab initio eukaryotic gene-finders.. Bioinformatics 2004, 20:2878-9.

5. Korf, I.: Gene finding in novel genomes.. BMC Bioinformatics 2004, 5:59.

6. Delcher, A. L., Harmon, D., Kasif, S., White, O. & Salzberg, S.L.: Improved microbial gene identification with GLIMMER.. Nucleic Acids Res 1999, 27:4636-41.

7. Stanke, M. & Morgenstern, B.: AUGUSTUS: a web server for gene prediction in eukaryotes that allows user-defined constraints.. Nucleic Acids Res 2005, 33:W465-7.

8. Pertea, G., Huang, X., Liang, F., Antonescu, V., Sultana, R., Karamycheva, S., Lee, Y., White, J., Cheung, F., Parvizi, B., Tsai, J. & Quackenbush, J.: TIGR Gene Indices clustering tools (TGICL): a software system for fast clustering of large EST datasets.. Bioinformatics 2003, 19:651-2.

9. Rice, P., Longden, I. & Bleasby, A.: EMBOSS: the European Molecular Biology Open Software Suite.. Trends Genet 2000, 16:276-7.

10. Allen, J. E. & Salzberg, S.L.: JIGSAW: integration of multiple sources of evidence for gene prediction.. Bioinformatics 2005, 21:3596-603.

11. Thompson, J. D., Higgins, D. G. & Gibson, T.J.: CLUSTAL W: improving the sensitivity of progressive multiple sequence alignment through sequence weighting, position-specific gap penalties and weight matrix choice.. Nucleic Acids Res 1994, 22:4673-80.

12. Florea, L., Hartzell, G., Zhang, Z., Rubin, G. M. & Miller, W.: A computer program for aligning a cDNA sequence with a genomic DNA sequence.. Genome Res 1998, 8:967-74.

13. Birney, E. & Durbin, R.: Using GeneWise in the Drosophila annotation experiment.. Genome Res 2000, 10:547-8.

14. Kurtz, S., Phillippy, A., Delcher, A. L., Smoot, M., Shumway, M., Antonescu, C. & Salzberg, S.L.: Versatile and open software for comparing large genomes.. Genome Biol 2004, 5:R12.

15. Benson, G.: Tandem repeats finder: a program to analyze DNA sequences.. Nucleic Acids Res 1999, 27:573-80.

16. Quevillon, E., Silventoinen, V., Pillai, S., Harte, N., Mulder, N., Apweiler, R. & Lopez, R.: InterProScan: protein domains identifier.. Nucleic Acids Res 2005, 33:W116-20.

17. Lowe, T. M. & Eddy, S.R.: tRNAscan-SE: a program for improved detection of transfer RNA genes in genomic sequence.. Nucleic Acids Res 1997, 25:955-64.

18. Hofacker, I. L., Fontana, W., Stadler, P. F., Bonhoeffer, S., Tacker, M. & Schuster, P.: Fast Folding and Comparison of RNA Secondary Structures.. Monatshefte f. Chemie 1994, 125:167-188.

19. Enright, A. J., Kunin, V. & Ouzounis, C.A.: Protein families and TRIBES in genome sequence space.. Nucleic Acids Res 2003, 31:4632-8.

20. Remm, M., Storm, C. E. & Sonnhammer, E.L.: Automatic clustering of orthologs and in-paralogs from pairwise species comparisons.. J Mol Biol 2001, 314:1041-52.

21. **The repeatmasker website [**http://www.repeatmasker.org]
