# Supplementary material for: High-throughput bioinformatics with the Cyrille2 pipeline system
Source: BMC Bioinformatics. 2008 Feb 12;9:96. doi: 10.1186/1471-2105-9-96 (PMC2268656; doi:10.1186/1471-2105-9-96)
Supplement: Additional file 3 — Fiers.Cyrille2.Suppl3.pdf contains the results of a test run with the pipeline from Figure 3 and 50 randomly downloaded BACs. [file 1471-2105-9-96-S3.DOC]

Supplementary Information 3 to:

# **High-throughput bioinformatics with the Cyrille2 pipeline system**

### **Mark WEJ Fiers^1^, Ate van der Burgt^1^, Erwin Datema^1^, Joost CW de Groot^1^, Roeland CHJ van Ham^1§^**

^1^Applied Bioinformatics, Plant Research International, PO Box 16, 6700AA Wageningen, The Netherlands

^§^Corresponding author

## **Results from a Cyrille2 test run**

The test run used the pipeline definition from Figure 3 and 50 randomly selected BAC sequences from *Arabidopsis* (4.8Mb).

| **Node** | **No. jobs** | **Object Type** | **No Generated** |
| --- | --- | --- | --- |
| Upload Fasta Dna | 1 | BAC | 50 |
| Genscan | 50 | Exon | 6,342 |
|  |  | PolyA | 979 |
|  |  | Promotor | 926 |
|  |  | Cds | 995 |
|  |  | Gene | 995 |
| GlimmerHMM | 50 | Exon | 6,493 |
|  |  | Cds | 1346 |
|  |  | Gene | 1346 |
| GeneId | 50 | Exon | 5,521 |
|  |  | Cds | 1,096 |
|  |  | Gene | 1,096 |
| SNAP | 50 | Exon | 7,029 |
|  |  | Cds | 1,713 |
|  |  | Gene | 1,713 |
| GeneSplicer | 50 | Donor site | 59,541 |
|  |  | Acceptor site | 69,911 |
| tRNAscan-SE | 50 | Exon | 20 |
|  |  | tRNA | 19 |
| MarScan | 50 | Mar | 858 |
| Tandem Repeat Finder | 50 | Repeat | 1,036 |
| BlastIf blastx | 50 | Blast Hsp | 41,451 |
|  |  | Blast hit | 16,094 |
|  |  | Raw output | 50 |
| GFFCds2Transcript | 5,150 | Transcript Seq | 5,150 |
| BlastIf blastx | 5,150 | Blast Hsp | 279,295 |
|  |  | Blast hit | 218,978 |
|  |  | Raw output | 5,150 |
| **Total** | **10,751** |  | **735,184** |
